# Radiological safety assessment of sugar consumption in South Africa—a study of ^226^Ra, ^228^Ra, and ^40^K levels

**DOI:** 10.3389/fpubh.2025.1534383

**Published:** 2025-01-29

**Authors:** Samuel Odumu Ogana John, Stephen Friday Olukotun, Moses Mpofana Radebe, Manny Mathuthu

**Affiliations:** ^1^Center for Applied Radiation Science and Technology (CARST), North-West University (Mahikeng Campus), Cnr Albert Luthuli Road and University Drive, Mmabatho, South Africa; ^2^Department of Physics and Engineering Physics, Obafemi Awolowo University, Ile-Ife, Nigeria

**Keywords:** activity level, effective dose, chronic daily intake, sugar, gamma spectrometry, South Africa, cancer risk

## Abstract

Human exposure to natural radionuclides in the environment primarily occurs through ingestion of foodstuffs, highlighting the importance of continuous monitoring of radionuclide levels in foodstuffs by ensuring consumer safety and compliance with regulatory standards. Using gamma spectrometry, this study investigates activity concentration levels of ^226^Ra, ^228^Ra, and ^40^K, and associated radiological health risks, in 14 commonly available sugar brands in South Africa. The activity concentration levels of these natural radionuclides ranged from 2.01 ± 0.13 to 7.93 ± 0.34 Bq/kg for ^226^Ra, 2.90 ± 0.10 to 7.09 ± 0.32 Bq/kg for ^228^Ra, and 209.40 ± 4.79 to 453.20 ± 10.49 Bq/kg for ^40^K. The respective mean values were 3.83 ± 0.21, 2.90 ± 0.21, and 320.26 ± 7.41 Bq/kg, with brown sugar having lower values than with white sugar. Annual effective ingestion dose from intake of ^226^Ra, ^228^Ra, and ^40^K, for infants (1–2 years), children (7–12 years), and adults (>17 years) ranged from 0.28 to 0.69, 0.32 to 0.82, and 0.12 to 0.30 mSv/year, respectively, and the respective mean values are 0.40 ± 0.11, 0.45 ± 0.14, and 0.17 ± 0.05 mSv/year. ^228^Ra contributed the largest proportion to ingestion dose (46–67%), indicating potential radiation risk to bones. Lifetime cancer risk ranged from 1.25 × 10^−8^ to 4.95 × 10^−8^ for ^226^Ra, 1.95 × 10^−8^ to 4.77 × 10^−8^ for ^228^Ra, and 5.53 × 10^−7^ to 1.19 × 10^−6^ for ^40^K, with a total mean of 8.96 × 10^−7^ ± 2.31 × 10^−7^. Total chronic daily intake due to ^226^Ra, ^228^Ra, and ^40^K ranged from 0.26 to 0.56 with a mean of 0.39 ± 0.10 (mg/kg-day). The activity concentration levels of the natural radionuclides are within the reference value while annual effective ingestion dose and lifetime cancer risks were below international permissible limits. This indicates that sugar in South Africa is radiologically safe and does not pose significant radiological health risks. In this study, the measured activity levels are comparable to those reported in other similar studies. This baseline study highlights the importance of continuous monitoring of radionuclide levels in foodstuffs, ensuring consumer safety, compliance with regulatory standards, and contributing to ongoing discussions on radiological health risks associated with dietary habits. Public health initiatives could consider offering guidelines for safe levels of consumables such as sugar, especially among vulnerable populations such as children.

## Introduction

Our natural environment that chiefly consists of the earth and the atmosphere is endowed with abundant natural and mineral resources, and is a source of radioactivity to which humans are constantly exposed. Naturally occurring radioactive materials (NORMs) are these sources of ionizing radiation, primarily consisting of the radionuclides from the ^238^U and ^232^Th decay series and ^40^K (1–3). These radionuclides enter the human body mainly through the ingestion of food and beverages, and by means of the inhalation of gas in the air. Therefore, it is necessary to maintain control over the levels of radioactivity in the environment, especially in foodstuffs. According to the United Nations Scientific Committee on the Effect of Atomic Radiation (UNSCEAR) report, the concentration of natural radioactivity in food typically ranges from 40 to 600 Bq/kg, which can pose a radiological health risk to vital body organs if not checked (1, 4–6). The long-term use of fertilizers and their by-products to boost crop production contributes to an increase in levels of ^40^K and ^226^Ra concentrations in the soil where sugar cane as raw material for sugar production is cultivated (7, 8). The presence of natural radionuclides in foodstuffs and sugars can increase the effective internal radiation dose, and the presence of ionizing radiation may cause various cancer risks. For instance, ^232^U and its progenies, such as ^226^Ra, ^222^Rn, ^210^Pb, and ^210^Po, are carcinogenic and can accumulate in human vital organs, such as the kidney, bone, and lung, while ^232^Th and the progenies, such as ^228^Ra, can accumulate in liver and skeleton, potentially increasing mortality rates (9–11). ^40^K with a natural abundance of 1.17 × 10^−4^, and specific activity of 2.6 × 10^8^ Bq/kg, is an essential mineral, which is uniformly distributed in the human body due to its uptake from the diet—about 0.18% in adults and 0.2% in children; therefore, it is under homeostatic control (12). For this reason, many international organizations, such as the Food and Agriculture Organization (FAO), World Health Organization (WHO), UNSCEAR, International Commission on Radiological Protection (ICRP), and International Atomic Energy Agency (IAEA), collaborate to establish global guidelines on acceptable levels of radioactivity in foods (1, 10, 13, 14).

The radionuclide sources in the human diet are primarily found in milk products, meat products, grain products, leafy vegetables, roots and fruits, fish products, drinking water, and especially those frequently consumed food products—vary in concentration from one region to the other. The radioactivity of these radionuclide sources in the human diet depend on the amount of intake (1, 12). Sugar is consumed directly, especially in beverages, as s sweetener, and is an important ingredient used in baking, food preservation, fermentation, balancing flavors, caramelizing, and so on. In the form of simple sugar, it primarily provides carbohydrates such as sucrose and is high in calories, supplying energy. Therefore, the importance of sugar in the human diet is enormous, and its safe consumption is critical. South Africa is a world-ranking producer of sugar, and its domestic consumption was expected to increase by 2% from 1.73 to 1.77 million metric tons in the 2022–2023 market year due to population growth (15). The region where sugarcane, the major raw material for sugar production, is grown—KwaZulu-Natal and Mpumalanga provinces—are characterized by geological variations with differing levels of natural radionuclides in the soil due to the presence of abundant mineral deposits. This is likely to affect the final product of sugar consumed, which needs to be determined using reliable methods (16, 17).

High-purity germanium (HPGe) detectors are employed in gamma-ray spectrometry, as a non-destructive analytical technique. This technique is one of those recommended by the IAEA and other global regulatory bodies. It has been widely used to measure the concentrations of natural radionuclides, such as ^226^Ra, ^228^Ra, and their progeny, as well as ^40^K and artificial radionuclides such as ^137^Cs in environmental samples, including foodstuffs (18). The method requires a simplified sample preparation without chemical separation and is applicable in identifying and quantifying radioactivity levels in a material with good precision. A standard source with the same geometry and matrix—as a sample being analysed—is used to characterize the detector, ensuring high sensitivity, reliability, and accuracy of measurement (18–20). In this study, this method is utilized in the analysis of natural radionuclides in this study.

In recent years, some research studies have reported on the activity levels of radionuclides in various foodstuffs (5, 7, 9, 17, 21–24), employing methods that may differ from those applied in this study. The activity concentration levels of the natural radionuclides measured were further used to estimate the radiological health risks associated with the ingestion of the studied foodstuffs, including the effective dose for various age categories, lifetime cancer risks, and organ-specific doses (14, 25). The results presented in various studies showed varying activity concentration levels and corresponding health risk parameters. This variability was attributed to several factors, including the rate of food and water consumption, the radionuclide transfer factor, as well as the concentration of the radionuclide (12, 24). Additionally, background levels, climate conditions, agricultural practices, and anthropogenic factors also played a role in influencing these levels (12, 14). However, little work has been done on the activity concentration levels in sugar, especially in South Africa, making this study a baseline research study.

The present study was conducted to evaluate the activity concentration levels of the NORM radionuclides (^226^Ra, ^228^Ra, and ^40^K), in two types of sugar consumed in South Africa, and to estimate the associated radiological health risks, such as annual effective ingestion dose (AEID), excess lifetime cancer risk (ELCR), and other related parameters. The study aims to evaluate compliance of radionuclide levels in sugar with international safety standards, such as those of IAEA, UNSCEAR, or WHO, while providing recommendations on safe sugar consumption levels based on the measured radiological risks. This will contribute to establishing a radiometric baseline standard database of natural radioactivity levels in South African foodstuffs, which can serve as a reference for possible future changes in activity concentrations in sugar. The South African food regulatory agency, as well as the sugar industry, will find this study helpful, as the results highlight the importance of monitoring radionuclide levels in food products to ensure consumer safety, compliance with standard regulations, and contribute to the ongoing discussion on radiological health risks associated with dietary habits and consumption.

## Materials and methods

### Sample and sample preparation

A notable 14 sugar samples, consisting of 4 brown sugars and 10 white sugars from different brands, were purchased from an open grocery store in Mahikeng—a North-West province of South Africa. These samples were selected to represent the most consumed sugars and are among the top 10 sugars in the country, based on a preliminary survey conducted prior to the sample collection (26, 27). The samples were brought to the laboratory, weighed, and placed into 500-ml polyethylene jars with screw lids, which were then sealed using adhesive tape. The jars were stored for 30 days to allow secular equilibrium to be reached between the parent ^226^Ra and its daughter radionuclides. The density of the samples ranged from 0.700 to 1.109 g/mL, and approximately 400 g of each sugar was prepared, with corrections made for differences in density and geometry. Each sample was assigned a unique identity based on its color type: SB for brown sugar and SW for white sugar.

### Experimental setup and sample analysis

The prepared sugar samples were each placed into a low-background, 11.50-cm thick, cylindrical lead-shielded casing of the detector to minimize background radiation. They were counted for 43,200 s. A Canberra p-type well High-Purity Germanium (HPGe) gamma spectrometer, model GCW2021 (Canberra Industries Inc., Detector Products, Meriden, USA), connected to a multichannel analyser (MCA) model DSA 1000 (Canberra Industries Inc., Detector Products, Meriden, USA), and using Genie 2000 (Canberra Industries Inc., Detector Products, Meriden, USA) vs. 3.3 Gamma Acquisition and Analysis software (Canberra Industries Inc., Detector Products, Meriden, USA), was employed in the sample measurement and spectrum analysis, respectively. Inside the detector shielding, the background radiation was measured using an empty jar of the same geometry as that of the sample. Prior to calculating the specific activity concentration, the background value was subtracted from the gamma radiation for each sample.

**Figure 1:**
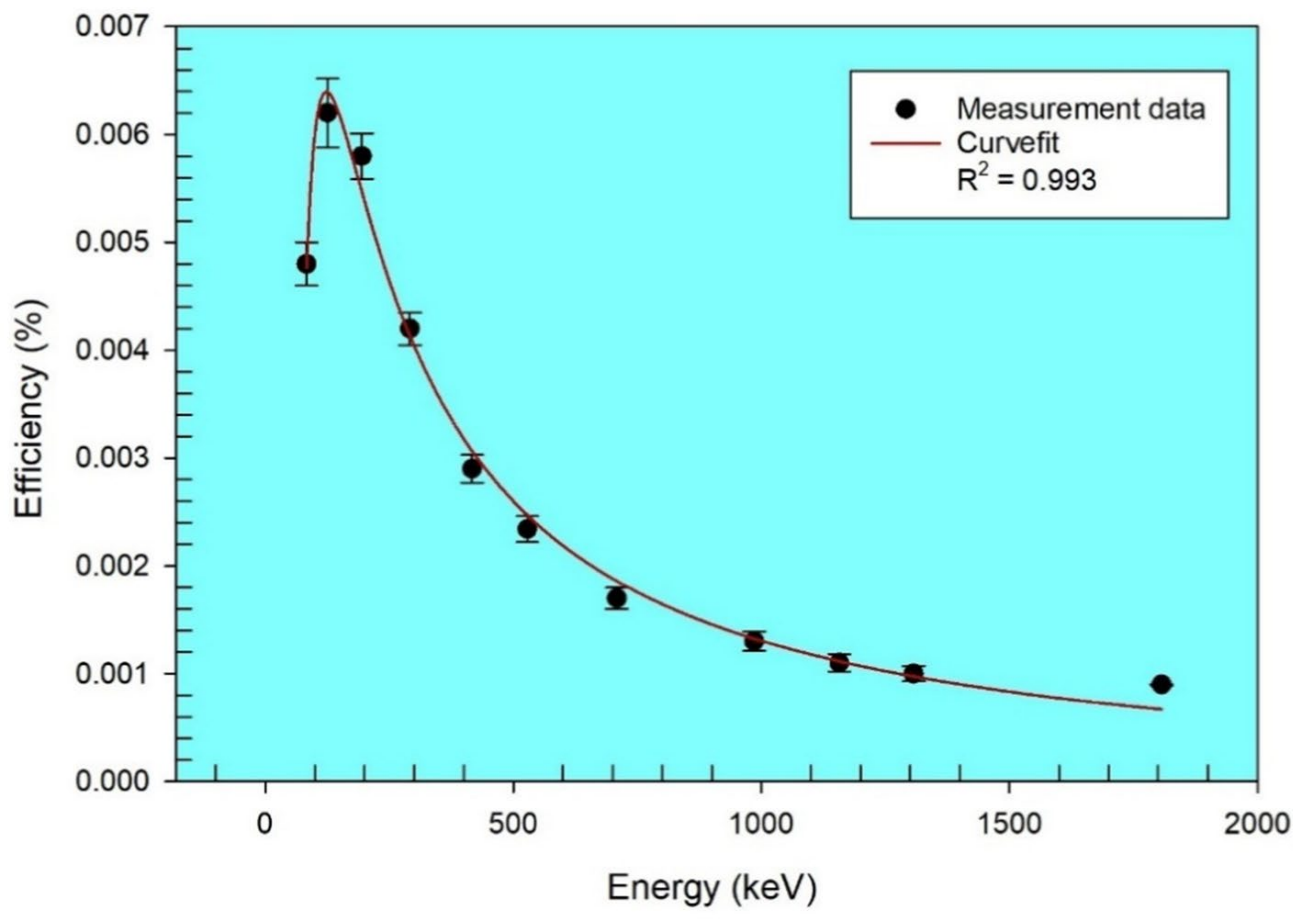
Efficiency– energy calibration curve for HPGe gamma detector.

The detector’s energy resolution was 1.7 keV full width at half maximum (FWHM) at 1.33 MeV of the ^60^Co gamma line, with a relative efficiency of 20%. The detector’s efficiency–energy calibration was performed using a multinuclide standard source, which is capable of emitting a wider range of gamma rays from 46.54 to 1836 keV. The multinuclide include ^210^Pb, ^241^Am, ^226^Ra, ^137^Cs, ^54^Mn, ^60,57^Co, and ^88^Y (18)—all homogeneously distributed in a container of similar geometry to that used for counting the samples. The curve for the efficiency–energy calibration is shown in Figure 1, with the fitting function given by the polynomial in Equation 1, at *R*^2^ = 0.993. Expanded HPGe gamma spectrometer calibration details are also provided in a previous study (28).


(1) $$\varepsilon \left(\%\right)=\exp\;\left(-186.00+127.60\ln\left(E\right)-34.95\ln{\left(E\right)}^{2}\;\right)$$

where ε(%) is the efficiency–energy calibration and E (MeV) is the corresponding photo peak energy.

The equipment was validated using IAEA-standardized reference materials, such as ^152^Eu, ^133^Ba, and IAEA-RGU-1, to ascertain the detector’s accuracy. The activity levels of the sources were measured using the detector and then compared to the certified values, adjusting for decay over time using the online Rad Pro decay calculator (Ludlum Measurements, Inc., Sweetwater, USA). The measured result for ^152^Eu (26.027 kBq) was in close agreement with the calculated activity (25.447 kBq), resulting in a percentage error of 2.281%, with a reference date of 1 December 2013. Compared with other published methods, HPGe gamma spectrometry, as used in this study, provides superior sensitivity, allowing for the detection of radionuclides at lower activity concentrations.

In this study, the limit of detection (LOD) and limit of quantification (LOQ) for the ^152^Eu were reported to be 0.16 and 0.48 Bq/kg, respectively, which compare favorably with LODs reported in similar studies ranging from 0.059 to 0.287 Bq/kg (20, 29). The method also demonstrated high accuracy, with relative standard deviation (RSD) of 0.0157, aligning with the precision of other gamma spectrometric studies. Analysis time was optimized to 43,200 s, which is competitive compared to other methods requiring longer sample preparation and counting times. To maintain the integrity of results and ensure compliance with regulatory requirements, the quality assurance standard method applied in this study is based on the guidelines provided in previous studies (18, 30).

Furthermore, in this study, the activity concentrations of both the radionuclides and their daughter radionuclides were determined through various gamma lines and those of. The gamma line peak energies used for ^226^Ra progenies included ^214^Pb (295.21 keV, Pγ = 19.2% and 351.95 keV, Pγ = 37.20%) and ^214^Bi (609.31 keV, Pγ = 46.30% and 1120.29 keV, Pγ = 15.10%). For ^228^Ra the gamma lines of its progenies, ^212^Pb (238.63 keV, Pγ = 44.60%), ^212^Bi (727.17 keV, Pγ = 11.80%), and ^228^Ac (911.60 keV, Pγ = 27.70% and 969.11 keV, Pγ = 16.60%) were used, while for ^40^K, its single gamma line at 1460.81 keV, Pγ = 10.67% was utilized (4, 13, 19). The weighted mean of the values of the progenies for the respective radionuclides were calculated, along with their uncertainties. The samples were measured at the Centre for Applied Radiation Science and Technology (CARST), North-West University (Mahikeng Campus), South Africa.

### Activity calculation

The specific activity concentration A_s_ (Bq/kg) for each of the natural radionuclides in the sugar samples was computed using Equation 2 while the minimum detectable activity concentration (MDA) (Bq/kg) was calculated using Equation 3 (4, 13, 19, 31), both were achieved through the Genie 2000 vs. 3.3 Gamma Acquisition and Analysis software.


(2) $$A_{s}\;\left(\mathrm{Bq}/\mathrm{kg}\right)=\frac{n_{s}-n_{b}}{P_{\gamma }\cdot \varepsilon_{\gamma }\cdot t\cdot m}$$

where n_s_ is the sample’s counts of a particular photopeak, n_b_ is the background count of a photopeak in the spectrum, P*_γ_* is the emission probability of gamma rays at the given energy photopeak, *ε*_γ_ is the detection efficiency, t is the time taken in seconds for the sample measurement, and m is the mass of sample in kilogram.


(3) $$MDA\;\left(\mathrm{Bq}/\mathrm{kg}\right)=\frac{2.71+4.65\;\sqrt{B}}{P_{\gamma }\cdot \varepsilon_{\gamma }\cdot t\cdot m}$$

where B is the background count for the gamma line under consideration, while other parameters remain the same as described in Equation 2. The minimum detectable activity for the radionuclides of interest computed at 5.00% MDA confidence factor is 1.34 Bq/kg for ^226^Ra, 1.83 Bq/kg for ^228^Ra, and 4.39 Bq/kg for ^40^K, respectively.

### Estimation of radiological health risks

The annual effective ingestion dose (AEID) is the radiation dose absorbed or received in a year by various body organs due to the ingestion of natural radionuclides present in food. Radiological risks from radiation exposure are described using this parameter, balancing the effects of carcinogens, life-shortening, and hereditary impacts; it is estimated using Equation 4 (29, 32)


(4) $$\mathrm{AEID}\;\left(\mathrm{mSv}/\mathrm{year}\right)=\overset{n}{\underset{i=1}{\sum }}\left(A_{S}\times C_{Ann}\right)\times I_{DC}$$

where $A_{S}$ is the specific activity concentration of the radionuclide of interest *i* (Bq/kg), $C_{Ann}$ is the mean annual consumption (kg/year) which is 30.7 kg/year (26) and $I_{DC}$ is the ingestion dose coefficient of radionuclide (Sv/Bq). $I_{DC}$ in Sv/Bq for ^226^Ra is 2.8 × 10^−7^, for ^228^Ra is 6.9 × 10^−7^, and for ^40^K is 6.2 × 10^−9^ (29, 33).

The annual effective ingestion dose E_D_ by age group of infants (age 1–2 years), children (age 7–12 years), and adults (age > 17 years) (33) due to ingestion of radionuclides present sugar, is estimated using Equation 5 (21). The idea of estimating age-dependent dose per unit intake is to extend the limited data on tissue concentrations while acquiring more broadly based dose estimates (1).


(5) $$E_{D}=A_{S}\;I\;F_{\mathrm{dc}}$$

where E_D_ is the annual effective ingestion dose, $A_{S}$ is the specific activity concentration of radionuclides, I is the annual sugar intake or consumption rate (10.23 kg/year for infants; 20.47 kg/year for children), and F_dc_ is the dose conversion factor by age group; the values are presented in Table 1 (1, 33).

**Table 1:** Dose conversion factors for ^226^Ra, ^228^Ra, and ^40^K used in determining annual effective dose for different age groups (1, 33).

Age group	Dose conversion factors (mSv/Bq)		
	^226^Ra	^228^Ra	^40^K
Infants (1–2 years)	9.6 × 10^−04^	5.7 × 10^−03^	4.2 × 10^−05^
Children (7–12 years)	8.0 × 10^−04^	3.9 × 10^−03^	1.3 × 10^−05^
Adults (>17 years)	2.8 × 10^−04^	6.9 × 10^−04^	6.2 × 10^−06^

Based on the ingestion exposure pathway, the excess lifetime cancer risk (ELCR) estimates the probability that an individual or population will develop cancer over a lifetime due to exposure to ionising radiation or potential carcinogens at a given rate. ELCR is estimated using Equation 6 (34, 35).


(6) ELCR=CDI×SF

where CDI is the chronic daily intake averaged over 70 years (mg/kg-day) which is determined using Equation 7, while SF is the slope factor expressed in (mg/kg-day) which represents the slope of the dose–response curve for cancer risk having values of 5.2 × 10^−6^ for ^226^Ra, 5.6 × 10^−6^ for ^228^Ra, and 2.2 × 10^−6^ for ^40^K (34), for the public and especially for stochastic effects. The slope factor translates the estimated daily intake, averaged over a lifetime of exposure, into an increased risk of an individual developing cancer.


(7) $$CDI=\frac{A_{S}\times I_{f}}{W_{B}}$$

where $A_{S}$ is the specific activity concentration of radionuclides, $I_{f}$ is the intake rate of the foodstuff, sugar, 30.70 kg/year, and $W_{B}$ is the body weight of an individual (in kg) which is usually assumed to be 70 kg for the adults (34, 35).

## Results and discussion

The results of the measured activity concentrations and uncertainties of the radionuclides ^226^Ra, ^228^Ra, and ^40^K in (Bq/kg) in 14 different brands of sugar consumed in South Africa are presented in Table 2. The results show that the radionuclides under study were detected in all the samples at varied concentrations. The specific activity concentration indicates that ^226^Ra activity varied from 2.01 ± 0.15 Bq/kg in sample SW-12 to 7.93 ± 0.14 Bq/kg in sample SW-11, with a mean value of 3.83 ± 0.21 Bq/kg. The ^228^Ra activity varied from 2.90 ± 0.18 Bq/kg in sample SW-12 to 7.09 ± 0.10 Bq/kg in sample SW-11, with a mean of 2.90 ± 0.21 Bq/kg, while ^40^K ranged from 209.40 ± 4.79 Bq/kg in sample SW-12 to 453.20 ± 10.49 Bq/kg in sample SW-11, with a mean of 320.26 ± 7.41 Bq/kg. The activity concentrations of the radionuclides measured are found to be below the worldwide recommended reference value set by the United Nations Scientific Committee on the Effects of Atomic Radiation (1) for the diet which is 22 Bq/kg for ^226^Ra and 15 Bq/kg for ^228^Ra. However, in this study, the activity concentration levels of ^226^Ra and ^228^Ra are found to be less than those reported by (9, 20, 22, 36), but greater than those of (37–39). However, the concentration of ^40^K in the body is under homeostatic control because it is uniformly distributed in the body due to its intake present in foodstuffs (1, 12), there is no specific reference level provided.

**Table 2:** Specific activity concentrations (Bq/kg) of NORMs in sugar samples consumed in South Africa.

Sample ID, radionuclides	Specific activity concentration (Bq/kg)			AEID (mSv/year)
	^226^Ra	^228^Ra	^40^K	
SB-1	2.43 ± 0.17	3.15 ± 0.19	249.10 ± 6.05	0.09
SW-2	4.18 ± 0.28	3.63 ± 0.13	400.80 ± 9.15	0.14
SW-3	4.01 ± 0.28	5.05 ± 0.32	426.60 ± 10.04	0.15
SB-4	3.43 ± 0.24	3.16 ± 0.18	248.70 ± 5.53	0.09
SB-5	2.73 ± 0.17	2.96 ± 0.19	255.90 ± 5.75	0.09
SW-6	2.59 ± 0.16	2.96 ± 0.19	257.30 ± 6.13	0.09
SB-7	2.51 ± 0.17	3.14 ± 0.20	254.70 ± 5.77	0.09
SW-8	4.79 ± 0.34	4.32 ± 0.26	328.40 ± 8.41	0.13
SW-9	3.65 ± 0.26	4.79 ± 0.29	386.10 ± 8.66	0.14
SW-10	3.33 ± 0.25	2.93 ± 0.28	373.20 ± 8.31	0.12
SW-11	7.93 ± 0.14	7.09 ± 0.10	453.20 ± 10.49	0.21
SW-12	2.01 ± 0.15	2.90 ± 0.18	209.40 ± 4.79	0.08
SW-13	2.56 ± 0.17	3.22 ± 0.19	252.40 ± 5.64	0.09
SW-14	7.49 ± 0.13	3.29 ± 0.13	387.80 ± 8.98	0.16
Mean	3.83 ± 0.22	3.76 ± 0.20	320.26 ± 7.41	0.12

**Figure 2:**
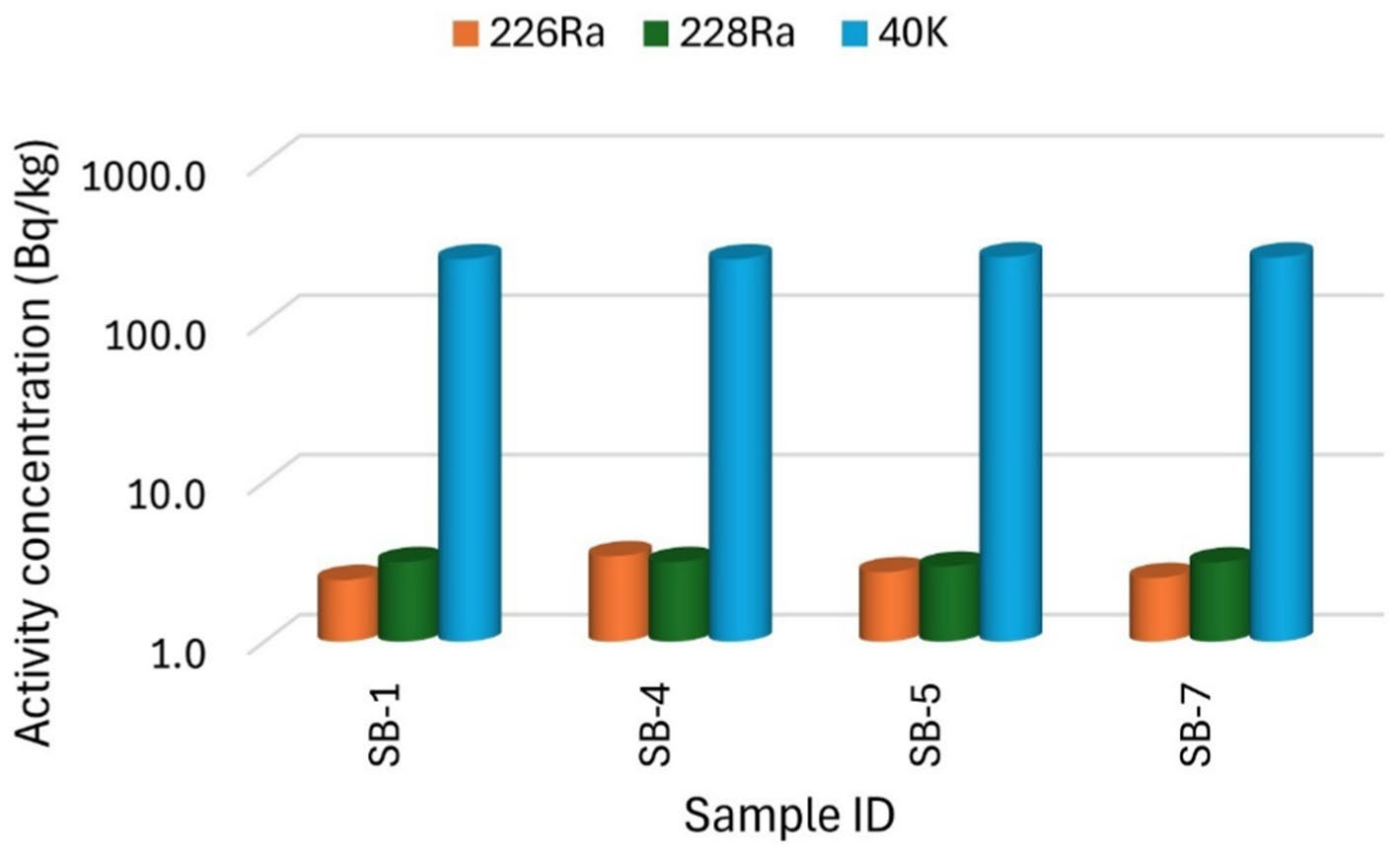
The specific activity concentration of radionuclides ^226^Ra, ^228^Ra, and ^40^K for the brown sugar samples.

**Figure 3:**
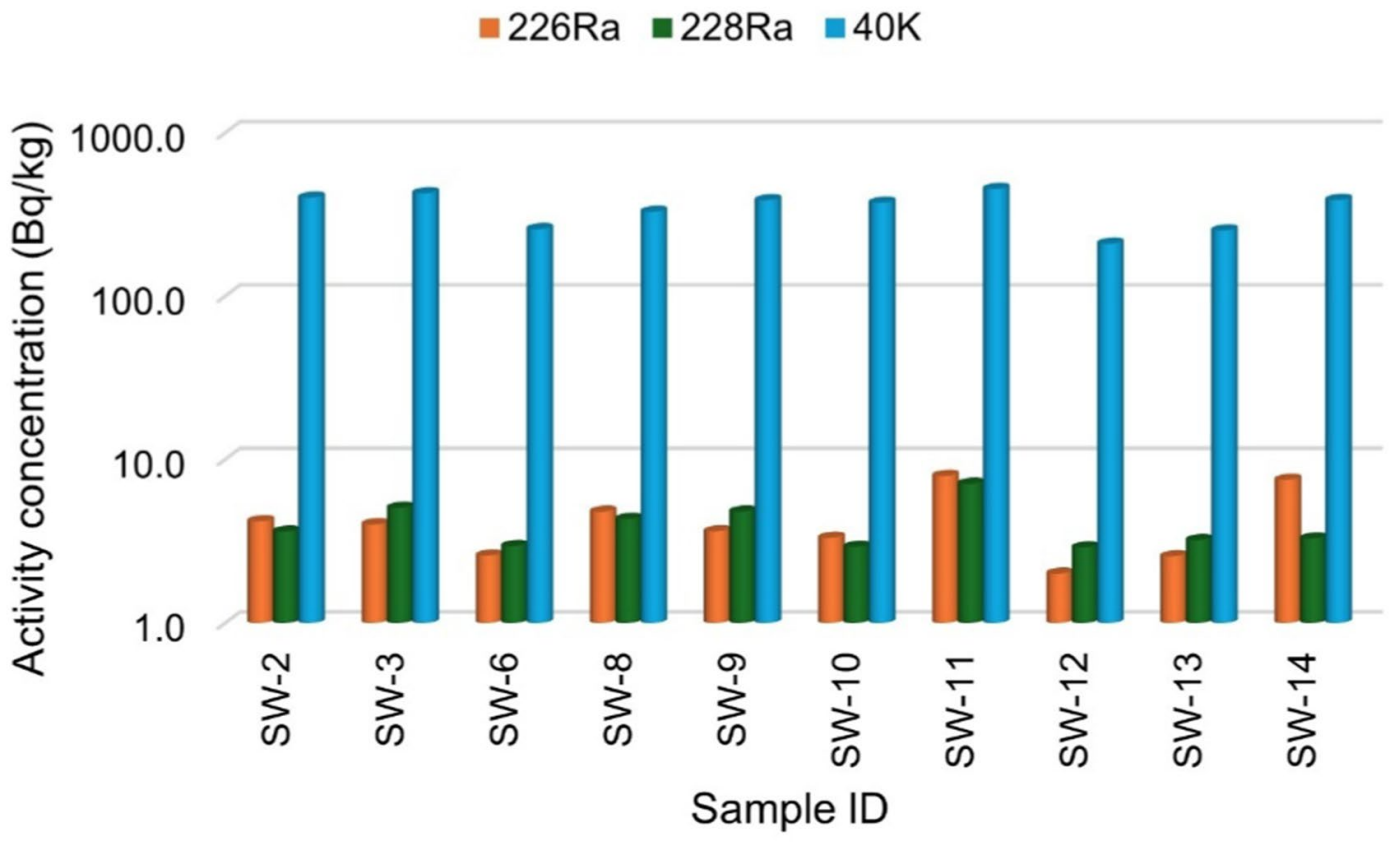
The specific activity concentration of radionuclides ^226^Ra, ^228^Ra, and ^40^K for the white sugar samples.

The distribution of the specific activity concentrations of the sugar samples shows that the activity concentration of ^40^K is higher in all the samples compared to the other radionuclides, following the order: ^40^K > ^228^Ra > ^226^Ra. The higher activity concentration of ^40^K in comparison to other radionuclides, reflects its natural abundance, with a specific activity of 2.6 × 10^8^ Bq/kg, which is greater than that of the other radionuclides (1). There is also variation in the activity concentrations among the samples studied, as shown in Figures 2, 3, with brown sugar samples displaying lower activity levels than white sugar samples. This variation may be attributed to the geological formation conditions and processes of the area where the raw materials were produced (40) and the industrial production processes. Notably, the activity concentration levels of the radionuclides in the sugar from this study are higher than those reported by sources (37–39), which could be due to regional variation.

Figures 2, 3 present the specific activity concentrations of the radionuclides ^226^Ra, ^228^Ra, and ^40^K in the brown and white sugar samples. Figure 2 shows the activity concentrations of radionuclides in brown sugar brands, while Figure 3 presents the concentrations of radionuclides in white sugar samples. Table 3 displays the comparison results of the range and mean activity concentrations of the radionuclides in the two types of the sugar samples. For ^226^Ra, the activity concentration range and mean values are 0.99 ± 0.08 Bq/kg and 2.78 ± 0.19 Bq/kg, respectively, for brown sugar samples, and 5.92 ± 0.22 Bq/kg and 4.25 ± 0.22 Bq/kg, for white sugar samples. For ^228^Ra, the activity concentration range and mean values are 0.19 ± 0.02 Bq/kg and 3.10 ± 0.19 Bq/kg, respectively, for brown sugar samples, and 4.20 ± 0.22 Bq/kg and 4.02 ± 0.21 Bq/kg, respectively, for white sugar samples. For ^40^K, the activity concentration range and mean values are 7.20 ± 0.52 Bq/kg and 252.10 ± 5.78 Bq/kg for brown samples, and 243.80 ± 5.70 Bq/kg and 347.52 ± 8.06 Bq/kg for white samples, respectively. The variation in the range and mean activity concentrations between the two types of sugar samples suggests that brown sugar has lower values than white sugar.

**Table 3:** Range and mean activity concentration values for the two sugar brands in this study.

Radionuclide	Brown sugar brand		White sugar brand	
	Activity range (Bq/kg)	Activity mean (Bq/kg)	Activity range (Bq/kg)	Activity mean (Bq/kg)
^226^Ra	0.99 ± 0.08	2.78 ± 0.19	5.92 ± 0.22	4.25 ± 0.22
^228^Ra	0.19 ± 0.02	3.10 ± 0.19	4.19 ± 0.22	4.02 ± 0.21
^40^K	7.20 ± 0.52	252.10 ± 5.78	243.80 ± 5.70	347.52 ± 8.06

The variation in the activity concentrations between the brown and white sugar samples could be related to the sources of the raw materials. Sugar cane—primarily produced in the KwaZulu-Natal and Mpumalanga provinces—is grown in areas with varying levels of natural radionuclides in the soil, which can affect the final product of sugar (16). Additionally, industrial refining processes and the presence of molasses may contribute to the variation between the different sugar sample types.

The estimated annual effective ingestion dose for the analysed sugar samples is presented in Table 2, column 5. The values obtained ranged from 0.08 to 0.22 mSv/year, with a mean value of 0.12 ± 0.04 mSv/year. These values fall below the recommended limit of 0.20–0.80 mSv (1, 14), suggesting that the levels of natural radionuclides in sugar samples are within a safe range for human consumption. Furthermore, these values are lower than those reported by a previous study (20), reinforcing the notion that the sugar products analysed in this study pose a minimal radiological risk to consumers. However, further analysis based on age variability, as outlined by UNSCEAR (1, 41) and using Equation 5, provided additional insight. Specifically, because the cells of children divide rapidly, they are more sensitive to radiation than adults, and as they live longer, chances are that cancer will have a reducing effect on their quality of life (42, 43), as presented in Table 4.

**Table 4:** Annual effective ingestion dose for different age groups (infants 1–2 years), children (7–12 years) and adults (>17 years) due to ^226^Ra, ^228^Ra, and ^40^K radionuclides in sugar samples in South Africa in this study.

Radionuclides in sugar	Description	Annual Effective Ingestion Dose (E_D_) (mSv/year)		
		Infants	Children	Adults
^226^Ra	Minimum	0.02	0.03	0.02
	Maximum	0.08	0.13	0.07
	Mean	0.04	0.06	0.03
^228^Ra	Minimum	0.17	0.23	0.06
	Maximum	0.41	0.57	0.15
	Mean	0.22	0.30	0.08
^40^K	Minimum	0.09	0.06	0.04
	Maximum	0.20	0.12	0.09
	Mean	0.14	0.09	0.06
	Accumulated minimum	0.28	0.32	0.12
	Accumulated maximum	0.69	0.82	0.30
	Accumulated mean	0.40	0.45	0.17
	UNSCEAR 2000 report	0.2–0.8	0.2–0.8	0.2–0.8

Table 4 presents the annual effective ingestion dose (E_D_) categorised by age group for individuals consuming sugar, focusing on the contributions from the radionuclides ^226^Ra, ^228^Ra, and ^40^K. The age groups considered include infants (1–2 years), Children (7–12 years), and adults (>17 years). For infants, the accumulated E_D_ values ranged from 0.28 to 0.69 mSv/year, with a mean of 0.40 ± 0.11 mSv/year, and are below the acceptable limit, indicating that sugar consumption for this group is radiologically safe. The accumulated E_D_ values for children varied between 0.32 and 0.82 mSv/year, with a mean of 0.45 ± 0.14 mSv/year. This range is below the recommended limits, suggesting that the risk for this age group is relatively low. For adults, the accumulated E_D_ values ranged from 0.12 to 0.30 mSv/year, with a mean of 0.17 ± 0.05 mSv/year, indicating a minimal risk associated with radiation exposure from sugar consumption. While all E_D_ values across the age groups are below the recommended limits, it is noteworthy that the maximum accumulated E_D_ for children exceeds the acceptable range limit by 2.50%. This finding suggests that children may be at risk of exposure to ionizing radiation through sugar consumption in South Africa. Therefore, it is essential to monitor and assess dietary intake of sugar in the vulnerable age group to mitigate potential health risks.

**Figure 4:**
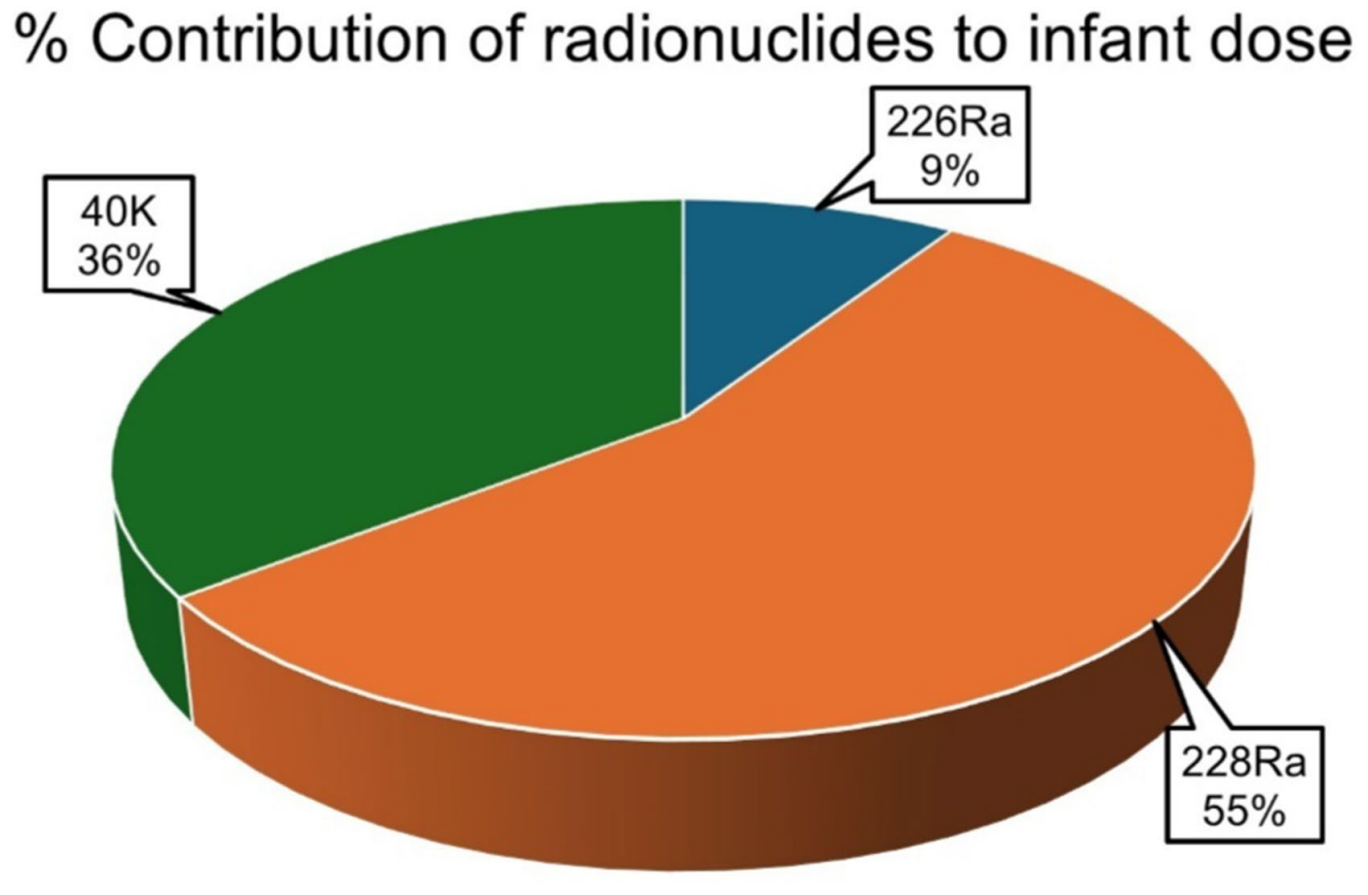
Percentage contributions of natural radionuclides ^226^Ra, ^228^Ra, and ^40^K to annual effective ingestion dose for the infants due to sugar consumption in South Africa.

**Figure 5:**
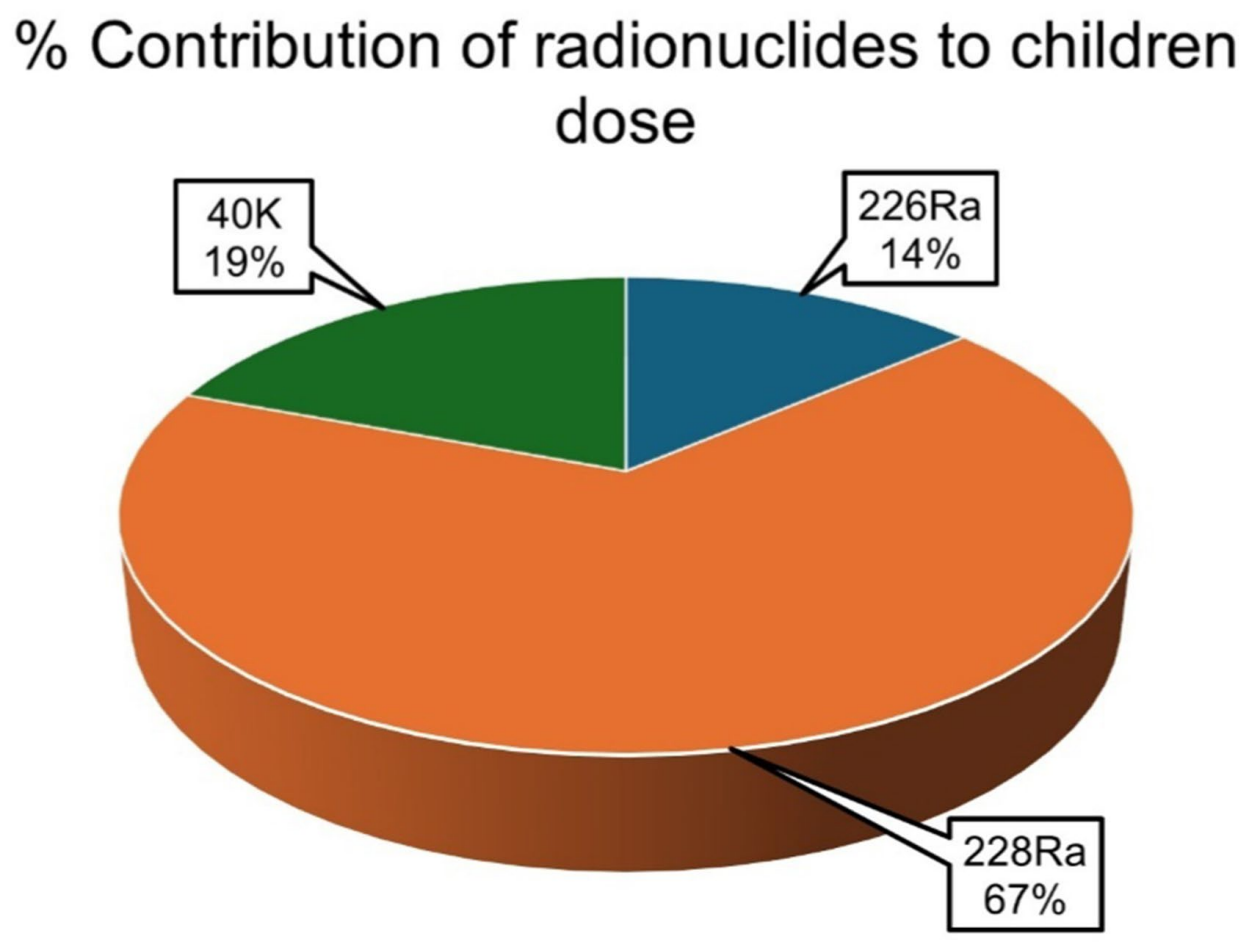
Percentage contributions of natural radionuclides ^226^Ra, ^228^Ra, and ^40^K to annual effective ingestion dose for the children due to sugar consumption in South Africa.

**Figure 6:**
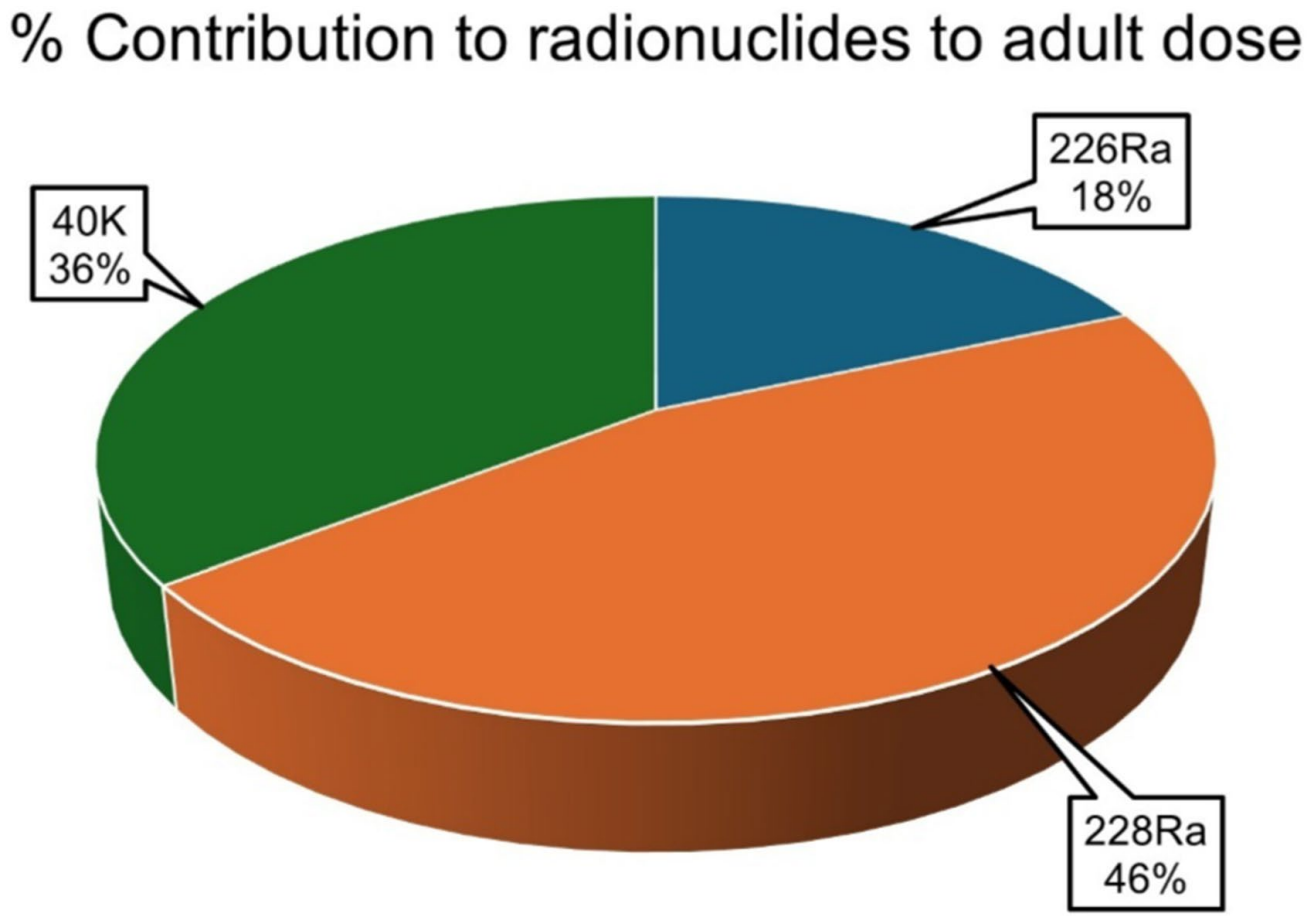
Percentage contributions of natural radionuclides ^226^Ra, ^228^Ra, and ^40^K to annual effective ingestion dose for the adults due to sugar consumption in South Africa.

The average percentage contribution of the individual natural radionuclides ^226^Ra, ^228^Ra, and ^40^K to the overall annual effective ingestion dose from the consumption of the sugar samples was computed, as shown in Figures 4–6. The average percentage contribution of ^226^Ra, ^228^Ra, and ^40^K in the sugar samples for the infants is 9, 55, and 36%, respectively (Figure 3); for the children is 14, 67, and 19% for ^226^Ra, ^228^Ra, and ^40^K, respectively (Figure 4); for the adults is 18, 46, and 36% for ^226^Ra, ^228^Ra, and ^40^K, respectively (Figure 5). The natural radionuclide ^228^Ra contributed the highest radiation dose to the sugar samples, with the higher contribution in the children category. ^226^Ra contributed the lowest dose, with the least contribution observed in the infant category. ^40^K lies between the two, with the highest value in both the infant and adult categories, consistent with the findings from (3, 41). The isotopes ^226^Ra and ^228^Ra tend to concentrate in the skeleton, replacing calcium in bones and teeth (1, 12). The findings in this study imply that, with ^226^Ra contributing the most to the radiation dose, there is risk of radiation damage to vital organs, such as bone marrow (10), highlighting the need for regular monitoring of foodstuffs. On the contrary, ^40^K is an essential element that is homeostatically controlled within human cells; therefore, its presence and determination are more related to physiological characteristics than dietary intake. Therefore, its contribution to radiation dose in sugar has less radiological health impact (1, 12). However, it is worth noting that the radionuclides ^226^Ra and ^228^Ra have been reported to contribute most significantly to the foodstuff dose, as indicated by previous studies (20, 21, 29).

**Figure 7:**
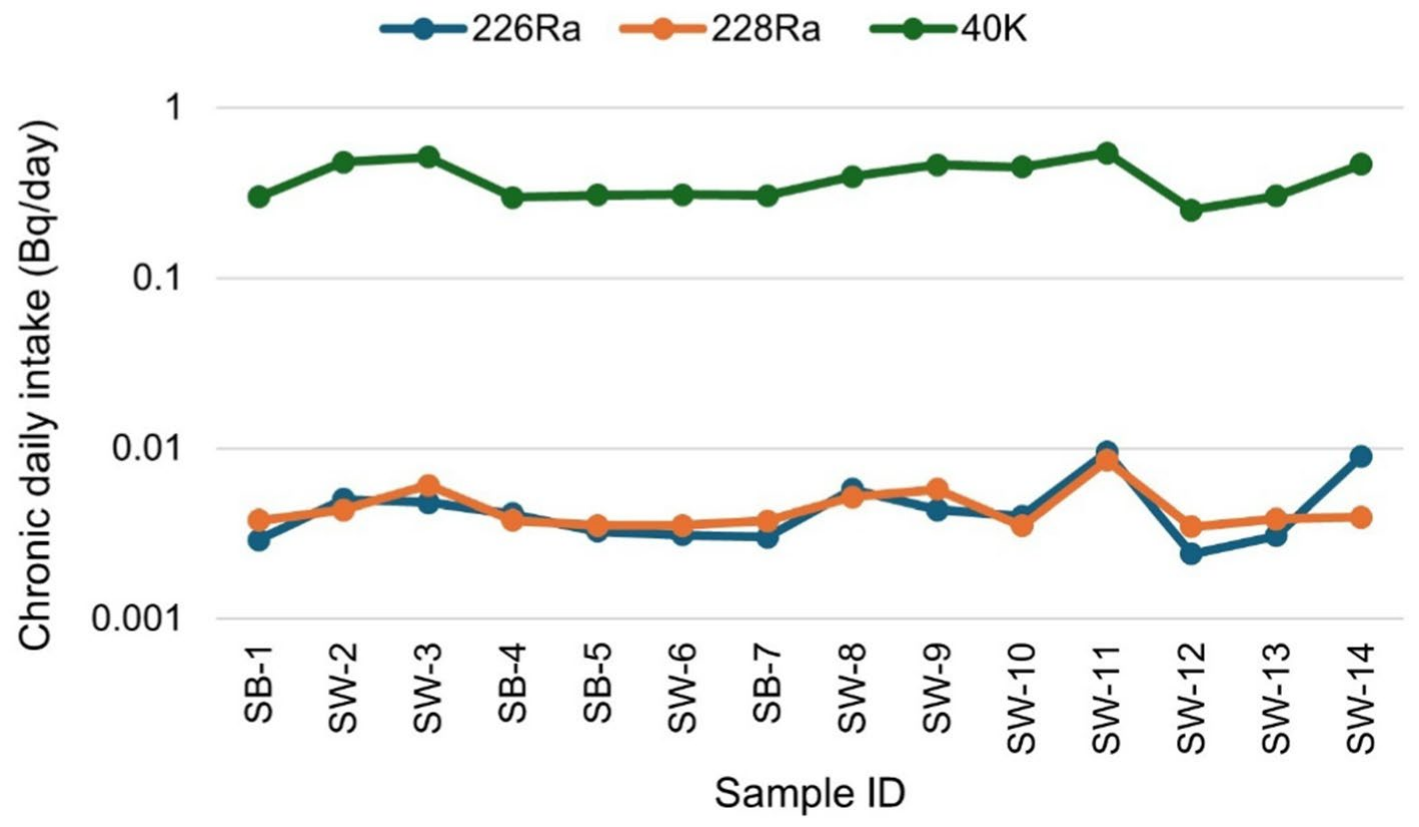
Chronic daily intake due to natural radionuclides ^226^Ra, ^228^Ra, and ^40^K for sugar consumption in South Africa.

Figure 7 presents the chronic daily intake (CDI) (mg/kg-day), averaged over a lifetime (usually assumed to be 70 years), of each contaminant (^226^Ra, ^228^Ra, and ^40^K) through the ingestion pathway via food consumption. The results show that the CDI for ^226^Ra varied from 2.41 × 10^−3^ to 9.52 × 10^−3^ with a mean of 4.60 × 10^−3^ ± 2.19 × 10^−3^. The CDI values for ^228^Ra ranged between 3.49 × 10^−3^ and 8.5 × 10^−3^, with a mean of 4.51 × 10^−3^ ± 1.44 × 10^−3^, while that of ^40^K varied between 0.25 and 0.54, with a mean of 0.38 ± 0.09. In summary, the total CDI ranged from 0.26 to 0.56, with a mean value of 0.39 ± 0.10. The CDI values in descending order are ^40^K > ^226^Ra > ^228^Ra, implying that sugar ingestion is associated with a high content of ^40^K.

**Figure 8:**
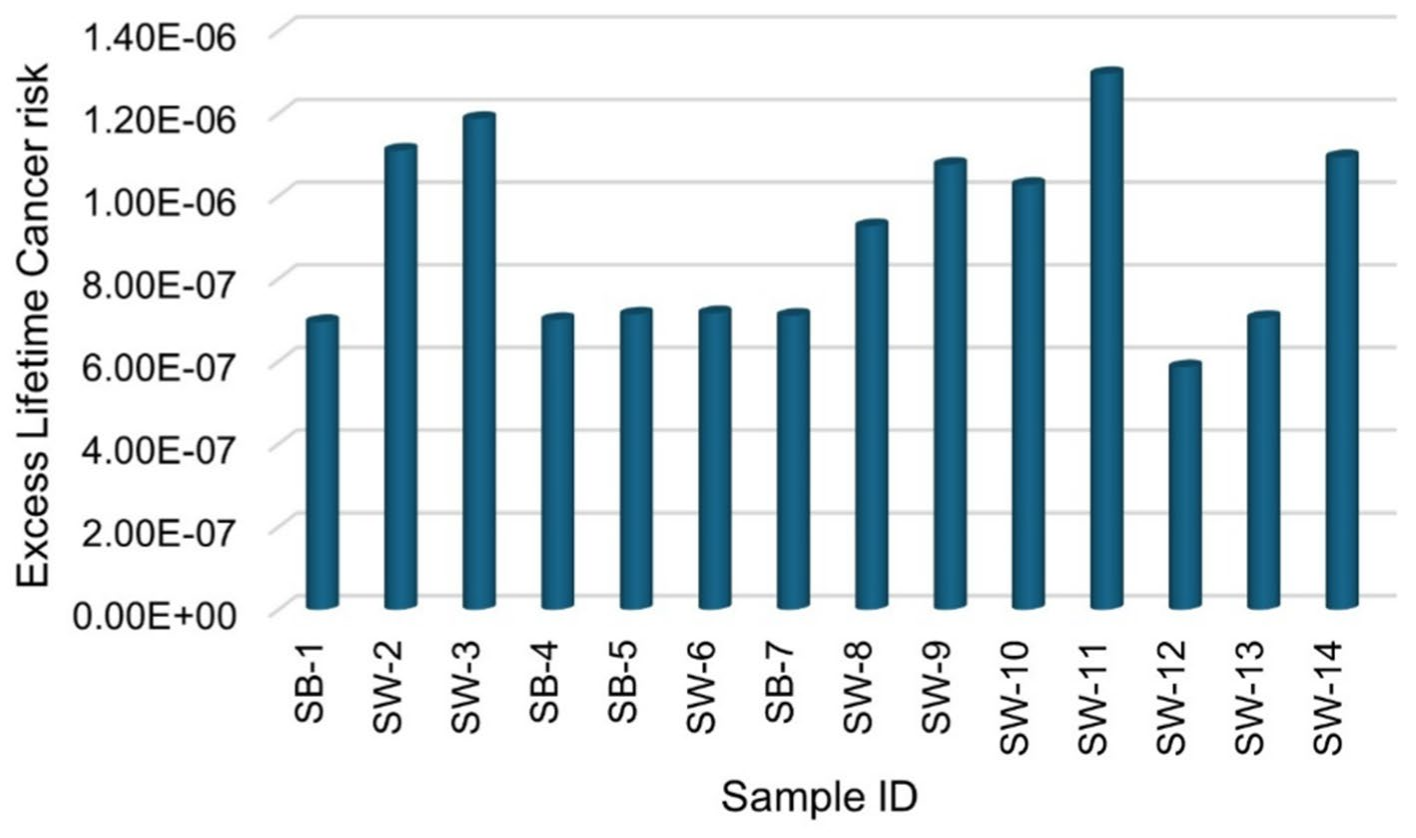
Total excess lifetime cancer risk due to natural radionuclides ^226^Ra, ^228^Ra, and ^40^K for sugar consumption in South Africa.

The excess lifetime cancer risk (ELCR) calculated in this study ranges from 1.25 × 10^−8^ to 4.95 × 10^−8^ with a mean of 2.39 × 10^−8^ ± 1.14 × 10^−8^ for ^226^Ra, from 1.95 × 10^−8^ to 4.77 × 10^−8^ with a mean of 2.53 × 10^−8^ ± 8.03 × 10^−9^ for ^228^Ra, and from 5.53 × 10^−7^ to 1.19 × 10^−6^ with a mean of 8.46 × 10^−7^ ± 2.16 × 10^−7^ for ^40^K, respectively. The total ELCR is presented in Figure 8, ranging from 5.86 × 10^−7^ to 1.30 × 10^−6^, with a mean value of 8.96 × 10^−7^ ± 2.31 × 10^−7^. This mean value is significantly below the acceptable ELCR threshold of approximately 1 × 10^−3^ (29), indicating that the radiological health risk associated with consuming sugar samples in this study is relatively low. Furthermore, the observed ELCR values are less than those reported by (25, 29), suggesting that the sugar products analysed in this study present a lower risk of inducting cancer over a lifetime than those previously assessed. This finding reinforces the conclusion that sugar samples consumed in South Africa are not only within acceptable radiological safety limits but also contribute minimally to long-term cancer risk, thereby supporting public health safety and dietary choices.

Table 5 presents the essential descriptive statistics for the natural radionuclides ^226^Ra, ^228^Ra, and ^40^K analysed in this study, including range, minimum, maximum, mean, standard deviation (STD), variance, skewness, and kurtosis. The mean values for these radionuclides are higher than their corresponding standard deviation values, indicating a high degree of uniformity in their distribution across the samples (40). The data exhibit positive skewness and kurtosis, suggesting that the distribution tails lean towards positive values. Notably, ^40^K has a negative skewness value and shows the highest variance among the radionuclides, indicating an uneven distribution of natural radioisotopes in the samples. This variance likely reflects the geological characteristics of raw material sources used in sugar production. Furthermore, the statistical analysis supports the observed order of activity concentration: ^40^K > ^232^Th > ^238^U.

**Table 5:** Statistical description of the specific activity concentration of the radionuclides ^226^Ra, ^228^Ra, and ^40^K in the sugar samples.

Radionuclides concentrations (Bq/kg)	The essential descriptive statistics							
	Range	Minimum	Maximum	Mean	STD	Variance	Skewness	Kurtosis
^226^Ra	5.92	2.01	7.93	3.83	1.82	3.32	1.52	1.58
^228^Ra	4.19	2.90	7.09	3.76	1.19	1.43	1.98	4.03
^40^K	243.80	209.40	453.20	320.26	81.67	6670.55	0.27	−1.63

Table 6 presents a comparative analysis of the mean activity concentrations of natural radionuclides (^226^Ra, ^228^Ra, and ^40^K) found in sugar samples and other related foodstuffs from this study and data from other countries, as well as global reference values provided by a previous study (1). From the results of this study, South Africa’s sugar samples exhibited a mean activity concentration of 3.83 ± 0.28 Bq/kg for ^226^Ra, 3.76 ± 0.20 Bq/kg for ^228^Ra, and a significantly higher concentration of 320.26 ± 7.41 Bq/kg for ^40^K. In comparison, Brazil showed a much higher concentration of ^226^Ra, at 21.91 ± 0.34 Bq/kg, but minimal levels of ^228^Ra (0.02 ± 0.58 Bq/kg) and ^40^K (0.66 ± 0.32 Bq/kg) (20). Iraq’s data indicates the absence of ^226^Ra measurements but reveals a substantial presence of ^226^Ra (5.92 ± 0.72 Bq/kg) and ^40^K (138.66 ± 0.83 Bq/kg) (9, 36), suggesting varying geological influences in these regions. Poland provides limited data, only reporting ^40^K at 4.58 Bq/kg (24), indicating relatively low levels of this radionuclide compared to this study. Other countries presented values comparable to those of this study while some are either less than or higher, accordingly (25, 37, 39, 44). When juxtaposed with the global reference values for foodstuffs, of 22 Bq/kg for ^226^Ra, and 15 Bq/kg for ^228^Ra, while no reference value for ^40^K (1), all the reported activity concentration values from this study and other countries are below the international thresholds for the natural radionuclides studied. This finding implies that the foodstuffs reported have low activity concentration levels, and their consumption is safe and may not cause exposure to radiation dose. In addition, based on these results, public health initiatives could consider offering guidelines for safe radiation levels in consumables such as sugar, especially for vulnerable populations such as infants and children.

**Table 6:** Comparison of mean specific activity concentration (Bq/kg) in sugar and related foodstuff samples from South Africa and other countries around the world.

Country	^226^Ra	^228^Ra	^40^K	Reference
South Africa	3.83 ± 0.28	3.76 ± 0.20	320.26 ± 7.41	Present study
Brazil	21.91 ± 0.34	0.02 ± 0.58	0.66 ± 0.32	(20)
Iraq	7.00	5.92 ± 0.72	138.66 ± 0.83	(9, 36)
Poland	–	–	4.58	(24)
Saudi Arabia	14.40 ± 1.39	–	–	(22)
Mali	1.10 ± 0.10	0.40 ± 0.04	< 0.31	(37)
Yemen	3.63 ± 0.25	2.03 ± 0.13	188.30 ± 6.03	(44)
Turkey	2.33 ± 0.20	1.69 ± 0.18	141.34 ± 10.45	(38)
Kenya	–	–	1.90 ± 0.20	(39)
Romania	4.58 ± 1.25	1.60 ± 0.79	21.53 ± 4.06	(25)
Worldwide value	22	15	–	(1)

## Conclusion

The activity concentration levels of the natural radionuclides ^226^Ra, ^228^Ra, and ^40^K in 14 brands of sugar samples from South Africa were determined using HPGe gamma spectrometry in this study. The results obtained were used to estimate the effective ingestion dose and lifetime cancer risk from sugar intake, based on models prescribed by UNSCEAR and USEPA. It was found that radioisotopes ^226^Ra, ^228^Ra, and ^40^K were detected in all the samples at varying concentrations, in the order of ^40^K > ^228^Ra > ^226^Ra, with brown sugar having lower values than white sugar. The activity concentrations of all the radionuclides in the sugar samples are below the reference values set by the United Nations Scientific Committee on the Effects of Atomic Radiation, indicating that consuming sugar is safe and may not cause radiation exposure. The analysis of the effective ingestion dose suggests values within the recommended limits, with children receiving the highest dose. Notably, ^228^Ra contributes the highest dose, which may pose a potential risk of radiation exposure to the bones. The total chronic daily intake due to the radionuclides is minimal, with ^40^K contributing the highest value. At the same time, the estimated excess lifetime cancer risk remains within the acceptable limit, indicating a lower radiological health risk associated with sugar intake. Additionally, the findings of this study are comparable to those reported in other countries.

Based on the evaluated health risk parameters, it can be concluded that consuming sugar from South Africa is safe and does not pose any significant radiological health risks. This study provides radiometric baseline data on natural radionuclides in sugar and the associated hazard indices. It also emphasizes the importance of continuously monitoring radionuclide levels in foodstuffs to ensure consumer safety, compliance with standard regulations, and to contribute to ongoing discussions about the radiological health risks of dietary habits. Further research is recommended to assess the long-term effects of radiation exposure on growth, development, and disease prevention, especially in children and infants. Additionally, examining the heavy metal content in sugar from South Africa would be valuable for a more comprehensive risk assessment.

## Data Availability

The original contributions presented in the study are included in the article/supplementary material, further inquiries can be directed to the corresponding author.
